# Understanding the Factors Driving Consumers’ Willingness to Pay for Gene-Edited Foods in China

**DOI:** 10.3390/foods13152348

**Published:** 2024-07-25

**Authors:** Shuqing Gao, Jingru Chen, Yuqin Yang, Guoyan Wang

**Affiliations:** School of Communication, Soochow University, Suzhou 215123, China; sqgao@suda.edu.cn (S.G.); chenjingru0919@163.com (J.C.); yangyuqin021@163.com (Y.Y.)

**Keywords:** gene-edited foods, willingness to pay, scientific literacy, social trust, technological perceptions

## Abstract

Gene editing contributes to enhancing food security through the creation of novel foods. However, public perception of gene-edited (GE) foods is crucial to their acceptance and adoption. This study expanded the knowledge–attitude–practice model and constructed an integrated framework comprising four dimensions: demographic factors, scientific literacy and beliefs, social trust, and perceptions of gene technology, aimed at explaining the public’s attitudes toward GE foods. A questionnaire survey was conducted (*N* = 649), revealing a positive attitude toward GE foods, with over 80% expressing a certain willingness to pay (WTP) for them. Factors such as income level, subjective knowledge, scientific beliefs, trust in scientists, trust in government, and trust in national technological capabilities and perceived benefits positively correlated with WTP. Conversely, objective knowledge, perceived risks, and perceived ethical concerns were negatively correlated with WTP. The impact of objective knowledge on attitudes toward GE foods demonstrated a significant, nonlinear relationship. Additionally, it is noteworthy that the Chinese public currently exhibits relatively low trust in national technological capabilities, necessitating vigilance against the emergence of conspiracy theories akin to those surrounding genetically modified foods. This research contributes theoretical insights into the public communication of GE foods.

## 1. Introduction

Gene editing is currently regarded as the forefront of theoretical advancements and an important area of research in bioethics [1]. Gene editing technology, specifically CRISPR/Cas, has rapidly advanced over the past two decades, allowing for its application in various organisms [2,3]. Gene editing can transform agriculture and shape the future of food production by improving crop yields and animal productivity [4,5,6], which can help guarantee food security for the growing global population [7].

Gene editing technology allows developers to make precise modifications to specific genomes, making it more cost-effective and efficient for novel crop development compared to genetic modification [8]. Gene editing technology can enhance crop yields and disease resistance, as well as increase the nutrient content of foods [9,10]. Currently, gene editing has been applied to various crops and foods, such as wheat, mushrooms, fruits, peanuts, and soybeans [11,12]. The application of gene editing technology in agriculture and food holds significant market value. A study indicated that the European Union could face a loss of over a trillion euros within ten years if this technology is not adopted [13]. Over the past few years, gene-edited (GE) foods have been introduced into the market, with several products being approved and sold in countries like the United States, Japan, and Canada [14,15]. In China, the development of GE foods is more complicated.

China has invested heavily in the research and development of GE crops [16,17] and has acquired numerous patents for this technology [13]. However, due to the regulatory framework and public acceptance, the Chinese government has been cautious about approving GE food products in China. The Chinese Ministry of Agriculture and Rural Affairs approved a safety certificate for GE soybeans enriched with oleic acid in 2023. This marks the first approval of gene-editing technology on a crop in China [15].

As a country with a large population, China accounts for 22% of the world’s population, while its arable land accounts for only 7%. Therefore, the need for crop improvement through biotechnology is urgent [18,19]. However, in the past 20 years, China has been plagued by a long-term debate over the safety of genetically modified (GM) foods, which has led to great difficulties in promoting GM foods in China [20,21,22]. From a technical standpoint, gene editing technologies do not involve inserting foreign genetic material (DNA) into organisms such as GE foods [23]. Therefore, compared with GM foods, public acceptance of GE foods may be higher [24,25]. However, considering the public has widely questioned GM foods, whether the public can accept GE foods remains to be explored further.

The success of food technology depends largely on the public’s interest in and attitude towards it [13,26,27,28,29]. Understanding public attitudes toward GE foods and the underlying influencing factors has emerged as pivotal topics within the academic and industrial realms [30,31,32,33]. Addressing these issues can offer valuable insights for policymakers and businesses. Public attitudes toward food technology are multifaceted, shaped by demographic factors, personal experiences, and technological perceptions [32,34,35,36].

In China, gene editing technology captured public attention following an incident involving gene-edited babies [37,38]. Scholars have started exploring the Chinese public’s perception and acceptance of GE foods [25,39,40]. However, there is presently a deficiency in a coherent theoretical framework that comprehensively examines the influence of diverse factors on individuals’ attitudes toward GE foods, particularly those associated with individual characteristics. Therefore, this study aims to establish a systematic explanatory framework for understanding the public’s attitude toward GE foods based on a comprehensive review of various influencing factors.

## 2. Literature Review

Gene editing, also called genome editing, is an intra-gene modification technology that enables the modification and transformation of specific genetic information within an organism [41]. Currently, gene editing technology has been increasingly applied in the food and agricultural industries, offering significant advantages over other biotechnological methods [7,42]. However, due to the complexity of gene editing technology and controversies in public opinion, there remains a certain skepticism among the public regarding GE foods [43,44].

### 2.1. Research Framework

According to the knowledge–cognition–attitude–behavior model (also known as the knowledge, attitude, and practices model) [45] and the Engel–Kollat–Blackwell model (EKB model) [46], individual attitudes are closely linked to personal knowledge and cognition, sequentially impacting individuals. Furthermore, in previous studies on food attitudes, demographic variables have often been treated as control variables. According to the EKB framework, however, demographic variables are crucial external factors influencing attitudes. In the orientation–stimulus–orientation–response model (OSOR model), which describes the occurrence of human attitudes and behaviors, demographic variables are more clearly regarded as the initial factors that affect people’s information processing [47,48,49].

**Figure 1:**
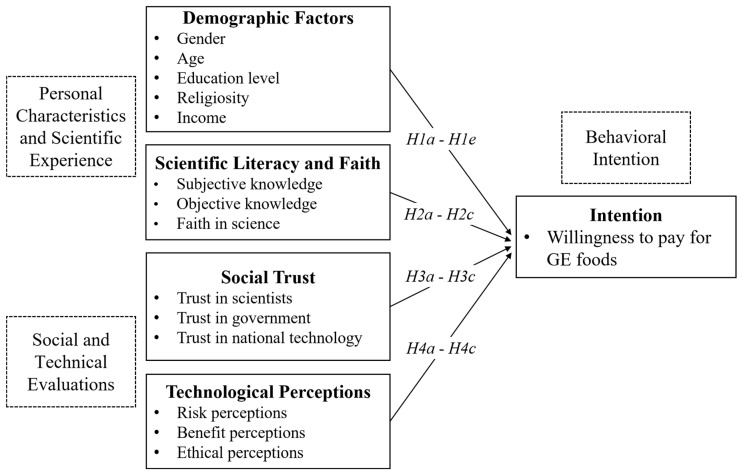
A theoretical model of explaining the public’s WTP for GE foods.

Therefore, by integrating these theoretical models, this study categorizes factors influencing public attitudes toward GE foods into four dimensions: demographic factors, scientific literacy and beliefs, social trust, and gene technology perceptions. In our theoretical model, demographic factors and scientific literacy characterize individual traits and scientific experiences. At the same time, social trust and perceptions of gene editing depict people’s cognitive processing of gene editing technology. Finally, this study selected willingness to pay (WTP) for GE foods as the behavioral indicator to measure public attitudes (the conceptual construction model is depicted in Figure 1).

### 2.2. Demographic Factors

According to the OSOR model, individual information processing is initially influenced by basic demographic and dispositional factors [47,50]. Therefore, demographic variables can be considered fundamental factors affecting individual attitudes towards food. Several studies have explored the impact of demographic variables on attitudes towards novel foods [51,52,53,54].

Various individual factors shape public attitudes toward GE food, with demographic variables typically exerting the apparent effects. Previous research indicates that, compared to women, men generally show greater support for gene editing technology [55]. Women prioritize food safety, leading to more negative attitudes toward genetically engineered foods [56]. Younger individuals usually exhibit higher acceptance of new technologies and are more willing to experiment with new products. Compared to older individuals, younger people express fewer concerns about GM and GE foods, displaying more positive attitudes [29,55,57,58].

The relationship between education level and attitudes towards gene technology remains inconsistent among researchers. Some studies suggest that higher education is associated with more negative attitudes towards gene editing technology [59]. In contrast, others find a positive correlation between education level and attitudes towards gene editing technology [60]. Additionally, some studies report no significant relationship between education and attitudes towards gene technology [61]. Concerning GE foods specifically, a study in Japan found that knowledge and educational attainment positively predict acceptance of GE foods [32].

Individuals with higher incomes commonly possess better access to information and material resources, enabling them to evaluate and handle potential risks more effectively [62]. Empirical research has consistently demonstrated a positive correlation between economic status and attitudes toward GE foods [63,64,65]. Religious beliefs often influence individuals’ moral perspectives, and religious adherents usually see gene editing technology as “playing God”. Therefore, individuals who hold firm religious beliefs generally express disapproval of the use of gene editing technology [64,66].

Not all gene technologies examined in the studies mentioned above are GE foods; for instance, some involve genetically modified organisms. Therefore, there remains a need for systematic exploration of the relationship between GE foods and demographic variables. Based on the analysis above, this study posits the following hypotheses:

**H1a:**  *Age is negatively related to people’s acceptance of GE foods.*

**H1b:**  *Men have a higher acceptance of GE foods compared to women.*

**H1c:**  *Educational level is positively related to people’s acceptance of GE foods.*

**H1d:**  *Income level is positively related to people’s acceptance of GE foods.*

**H1e:**  *Religiosity is negatively related to people’s acceptance of GE foods.*

### 2.3. Scientific Literacy and Scientific Beliefs

Scientific literacy is a crucial factor influencing people’s attitudes towards science and technology [67,68,69]. Some scholars have proposed the “information deficit model”, suggesting that increasing public scientific literacy can effectively improve negative attitudes toward technology [70,71,72]. In the context of gene editing technology, some studies have found a positive correlation between scientific knowledge and attitudes toward gene editing [64,66,73,74]. For example, compared to the general public, molecular biologists perceive higher benefits and lower risks associated with GE crops [75]. Other studies have further classified knowledge into subjective and objective categories, separately examining their relationships with attitudes toward GM foods [76,77,78]. Research indicates that objective knowledge exhibits a stronger correlation with attitudes compared to subjective knowledge [76]. Although many studies have supported the deficit model, some studies have found a negative correlation between scientific knowledge and attitudes toward technology [57,62]. Hence, more evidence is needed to determine whether scientific knowledge and beliefs influence the public’s attitudes toward GE foods.

Scientific belief assesses individuals’ trust in scientific principles and methods as reliable means of comprehending the world [79]. This belief is closely associated with both scientific literacy and attitudes towards technology [74,80,81]. Individuals with stronger scientific beliefs typically demonstrate higher acceptance of advanced technologies; for example, research has shown that those with robust scientific beliefs tend to express more positive attitudes toward gene editing technology [74].

Based on the above analysis, this study proposes the following hypotheses:

**H2a:**  *Subjective knowledge is positively related to people’s acceptance of GE foods.*

**H2b:**  *Objective knowledge is positively related to people’s acceptance of GE foods.*

**H2c:**  *Belief in science is positively related to people’s acceptance of GE foods.*

### 2.4. Social Attitudes Related to Gene Editing

The advancement and implementation of science and technology require the involvement of experts and institutions. Attitudes toward these individuals and organizations can influence the public’s technology acceptance [82,83,84]. Existing research indicates that governments serve as the primary channel for the public to access information about genetic technology [85]. Therefore, public trust in genetic technology largely depends on the level of trust in governance institutions [86]. In terms of GE foods, the higher the trust in food supervision institutions, the more likely individuals are to accept GE foods. Moreover, the acceptance of GE foods is closely related to the degree of trust in scientists [87]. This could be because the general public’s perception of complex technologies is often assessed using heuristics, which means it relies heavily on trust in the experts conducting the research and the institutions overseeing it [88].

One significant reason for the negative attitudes of the Chinese public towards GM foods is the spread of conspiracy theories. This conspiracy theory suggests that GM foods are biological weapons targeting China, and foreign countries do not consume GM foods [20]. This reflects public skepticism about the country’s level of biotechnological development. Therefore, trust in the national genetic editing technology capabilities (trust in national technology) may also be a crucial psychological factor influencing Chinese public attitudes toward GE foods.

One significant factor contributing to the Chinese public’s unfavorable perception of GM foods is the propagation of a conspiracy theory. The conspiracy theory posits that GM foods are biological weapons aimed at China by foreign entities that do not consume these foods themselves [15]. This reflects the public’s perception that their country lacks advanced scientific capabilities to identify such issues. Therefore, trust in national genetic technology may also be a crucial psychological factor influencing the Chinese public’s attitudes toward GE foods.

Based on the above analysis, this study proposes the following hypotheses:

**H3a:**  *Trust in scientists is positively related to people’s acceptance of GE foods.*

**H3b:**  *Trust in government is positively related to people’s acceptance of GE foods.*

**H3c:**  *Trust in national technology is positively related to people’s acceptance of GE foods.*

### 2.5. Gene Editing Perceptions

GE foods represent a novel category of food products, and public perception of these products significantly influences their attitudes. Generally, people’s perceptions of novel foods encompass evaluations of perceived risks, benefits, and ethical concerns [43,89,90,91]. However, there is currently inadequate public understanding of these perceptions associated with GE foods.

The potential benefits of GE foods are a key factor influencing public acceptance [92]. Perceptions of these benefits vary across cultures [36], with previous research indicating that higher perceived benefits of food correlate with greater individual acceptance [32].

Due to the disruptive nature of new technologies and the uncertainty of the technology itself, the public is generally concerned about new food products [93]. Previous studies have found that the higher the risk of gene editing technology perceived by individuals, the less supportive they are of its application [73,94].

Ethical perception involves an individual’s psychological assessment of whether a technology violates societal ethics or natural order, often measured with moral judgments [95,96]. These ethical perceptions significantly influence people’s attitudes toward novel foods and the application of gene editing technologies [74,97,98]. Research indicates that individuals have an innate resistance to scientific elements perceived as contradicting the natural order of things, which leads to public opposition to gene editing technology [74,99].

Based on the above analysis, this study proposes the following hypotheses:

**H4a:**  *Risk perception is positively related to people’s acceptance of GE foods.*

**H4b:**  *Benefit perception is positively related to people’s acceptance of GE foods.*

**H4c:**  *Ethical perception is positively related to people’s acceptance of GE foods.*

## 3. Materials and Methods

### 3.1. Design and Participant Recruitment

Credamo (www.credamo.com) is a proficient data platform that possesses a sample database comprising over 1.5 million participants [100], guaranteeing that our samples are sourced from various regions across the nation. Participants were randomly recruited nationwide in China through the Credamo online questionnaire platform from July to August 2022. The data collection targeted Chinese citizens aged 18 and above, and all participants provided informed consent. The predetermined number of questionnaires to be distributed was 700; those who did not provide serious answers (as indicated by their failure to answer attention-check questions) were excluded. The final dataset comprised 649 valid samples, including 238 male and 411 female participants (Table 1). The mean age was 30.21 years (SD age = 8.20 years).

### 3.2. Measures

#### 3.2.1. Attitudes toward Gene Editing Foods

A single question was used to measure attitudes toward human gene-editing foods (“I’m willing to pay for GE foods that are certified safe”). Responses were provided on a 7-point scale, with higher scores indicating more positive attitudes.

#### 3.2.2. Demographic Variables

Five demographic variables were collected in this study, including gender (0 = male; 1 = female), age, self-reported education level (years of schooling), religion (0 = not religious; 1 = religious), and self-rated income level (from 0 = very poor to 10 = very well off).

#### 3.2.3. Scientific Literacy and Scientific Beliefs

Objective knowledge. Given the absence of established measuring instruments for evaluating the general population’s objective knowledge about gene editing. This study used five questions adapted from previous studies [76,101] and supplemented them with a self-constructed question to evaluate participants’ proficiency in the principle of gene editing technology (e.g., “gene editing does not produce side effects due to off-target effects” and “You can tell if a fruit has been GE by its appearance”). Participants were asked to make a “right or wrong” judgment on the above questions and were awarded one point for each correct answer. Although the reliability coefficient for these 12 items is above 0.6 based on previous studies [102,103,104] is still at an acceptable level.

Subjective knowledge. The subjective knowledge of gene editing was assessed on a scale of 1 (indicating very limited knowledge) to 7 (indicating extensive knowledge) by a single self-assessment question, as indicated by prior research [31,78].

Belief in science. Five questions were borrowed from prior research to measure belief in science [95]. Participants evaluated the degree of alignment between each question statement (e.g., “science tells us everything we need to know about reality”) on a 7-point scale (from 1 very inconsistent to 7 very consistent). Cronbach’s α coefficient for this scale was 0.77.

#### 3.2.4. Social Attitudes Related to Gene Editing

Trust in national technological capabilities for gene editing (trust in national technology). Participants’ trust in national gene technology was measured by a question: “how much confidence do you have in our country’s level of science and technology in the field of gene editing compared to the rest of the world?”. Participants evaluated the magnitude using a rating scale ranging from 1 (“Very unconfident”) to 7 (“Very confident”).

Trust in national supervision of gene editing research (trust in government). The trust in national supervision of gene editing research was assessed on a scale of 1 (“Very unconfident”) to 7 (“Very confident”) by a single self-assessment question (“in respect of gene editing, I believe the relevant government departments will monitor the proper use of gene editing technology”).

Trust in scientists. Participants’ trust in gene editing researchers was assessed on a scale of 1 (“definitely no”) to 7 (“definitely yes”) by a single self-assessment question (“I think that our scientists deserve to be trusted in research in gene editing”).

#### 3.2.5. Perceptions of Gene Editing Technology

Risk perceptions. Six questions (e.g., “I think the risks of gene editing technology are difficult to control” and “I think it will take time for the risks of gene editing to manifest) were used to measure participants’ risk perception of gene editing, including the unknown risk dimension (unobservable, unfamiliar, and has delayed consequences) and dread risk dimension (uncontrollable, fatal, inequitable, putting future generations at risk) based on previous scholarly theorizing about the risks of gene editing technology [34]. Responses were measured on a 7-point scale from 1 = “not at all likely” to 7 = “very likely.” The Cronbach’s α coefficient of this scale was 0.88.

Benefit perceptions. We used two questions to measure the perceived benefits of gene editing technology in promoting social progress and preventing social regression. Responses were measured on a 7-point scale from 1, “definitely no”, to 7, “definitely yes”. The Cronbach’s α coefficient of this scale was 0.73.

Ethical perceptions. We used two questions to measure the public’s ethical perceptions of gene editing for general use. The study tasked participants with evaluating the unethical aspects of various gene editing technology usage scenarios (“gene editing research on non-human species”, “creation of animals or plants with new characteristics through genetic editing techniques”). The Cronbach’s α coefficient of this subscale was 0.69. All responses were recorded on a 6-point scale (1 = not at all morally wrong, 6 = extremely morally wrong), referring to previous moral judgment studies [105], with higher scores indicating a more negative moral outlook. It is important to note that a 1 in response means that people consider the behavior to be unethical and acceptable. In contrast, a score of 2–6 means that people consider the behavior to be unethical.

## 4. Results

### 4.1. Descriptive and Correlational Analysis

The results of descriptive statistics and correlations among the included variables are presented in Table 2.

The results showed that people exhibited relatively high subjective genetic knowledge (4.41 on a 7-point scale). In terms of participants’ objective genetic knowledge scores, the average correct responses were 8 out of 12 objective questions (M = 0.67, SD = 0.19), with 73.7% of participants answering more than half of the questions correctly. Most (92.1%) do not consider GE foods and GM foods inedible. However, participants’ understanding of gene editing remains incomplete. Compared to knowledge about genetic modification, awareness of gene editing among the public is relatively limited. For instance, approximately one-third of respondents believe that gene editing will not result in side effects due to off-target effects (see Table A1—Appendix A for details).

Public trust in the nation’s gene technology, trust in gene editing scientists, trust in government supervision of gene editing, and perceptions of benefits all received relatively positive ratings (score > 4). However, there is also a relatively high perception of risk associated with gene editing (M = 4.15, SD = 1.28), indicating a complex psychological perception of GE foods among the public.

In addition, people perceived the general use of gene editing to be less immoral (M = 2.69, SD = 1.36), with 24.3% believing that gene editing technology had nothing to do with ethics at all. About 16% (16.2%) of the participants considered using gene editing technology for non-humans to be a relatively severe ethical issue.

In terms of attitudes, participants exhibited a relatively high willingness to purchase gene-edited foods (M = 5.19, SD = 1.16), demonstrating a generally positive attitude towards them. 80.9% of participants expressed a relatively positive willingness to purchase GE foods.

Correlation analyses among the variables showed that the variables, including income, subjective knowledge, objective knowledge, belief in science, trust in scientists, trust in government, trust in national technology, and benefit perceptions, were positively correlated with attitudes toward GE foods, respectively. Objective knowledge, risk perceptions, and ethical perceptions were negatively correlated with attitudes toward GE foods, respectively. In addition, gender, age, educational level, and religiosity were not significantly related to attitudes toward GE foods, respectively.

### 4.2. Hierarchical Regression Analyses

To further reveal the independent effects of each variable on GE foods, this study utilized a hierarchical regression model in its analytical process. The result of the hierarchical regression analysis of the attitudes towards GE foods is shown in Table 3.

In Model 1, income had significant positive effects on attitudes towards GE foods (*β* = 0.20, *p* < 0.01). In Model 2, subjective knowledge (*β* = 0.12, *p* < 0.05), objective knowledge (*β* = −0.08, *p* < 0.05), and belief in science (*β* = 0.32, *p* < 0.01) were found to be significant factors that can predict attitudes towards GE foods. In Model 3, trust in scientists (*β* = 0.22, *p* < 0.01), trust in government (*β* = 0.24, *p* < 0.01), and confidence in national technology (β = 0.13, *p* < 0.01) were significant predictors. In Model 4, risk perception (*β* = −0.15, *p* < 0.01), benefit perceptions (*β* = 0.24, *p* < 0.01), and ethical perceptions (*β* = −0.10, *p* < 0.05) were found to be significant factors that can predict attitudes towards GE foods.

## 5. Discussion

### 5.1. Results and Discussion of Findings

In terms of research findings, this study identified a significant relationship between income and WTP for GE foods. Consistent with previous research, individuals with higher incomes exhibit a greater willingness to purchase and eat GE foods [25,30,64]. In this study, participants with higher economic status perceived lower risks and higher benefits associated with gene-editing technology, aligning with previous theoretical analyses [106]. Additionally, this study revealed a negative correlation between economic status and ethical perceptions, indicating that individuals with higher incomes tend to perceive GE technology as less morally wrong. This expands the theoretical framework linking economic status to attitudes toward gene-editing technology. Previous research has shown that individuals with lower economic status uphold stricter moral standards and are more prone to perceiving ethical dilemmas [107]. Therefore, the influence of economic status on attitudes toward GE foods may also be intertwined with moral cognition.

In terms of gender, some studies have found that females are more likely to accept GE foods [25], while others indicate that females show lower acceptance levels toward GE foods [25,108]. However, gender was not significantly associated with attitudes toward GE foods in this study. Previous research has suggested that females with children are less accepting of GE foods compared to those without children [109]. Therefore, the conflicting findings in these studies may be influenced by sample characteristics. Currently, GM foods are not yet on the market in China. It is possible that gender effects may emerge with the promotion of GE foods in the future. In terms of age, previous research has shown that younger individuals tend to have more positive attitudes toward GE foods [25,108,109]. Additionally, studies have indicated that age influences willingness to consume GE foods but not avoid purchasing them [30,110]. The current study found no significant relationship between age and attitudes toward GE foods.

Previous studies conducted in Europe and America have indicated that religious beliefs negatively influence attitudes toward gene editing technology, as individuals with religious beliefs tend to favor natural creation over researchers playing God to create new species [66,87,101,111]. However, this study found that religious beliefs do not significantly impact attitudes toward GE foods in China. Approximately 10.6% of participants in this study reported having religious beliefs. This is because China is a highly secular country with a religious population of about 14% [112]. Among Chinese religious adherents, Buddhism is the most prevalent [113], and prior research has shown that Buddhists are more accepting of gene editing technology than Christians, who tend to hold the most negative attitudes toward it [60,114]. This could potentially explain why, in China, there is no significant negative correlation between individuals’ religious beliefs and their attitudes toward GE foods. Additionally, in this study, education level showed no significant association with attitudes toward GE foods, consistent with some previous research findings [30,110]. However, other studies suggest that individuals with higher education levels are more likely to accept the application of gene editing technology [25,60]. Education is crucial in acquiring knowledge and understanding of gene editing technology [115], which may have important implications for supporting GE foods. Further research is needed to delve deeper into the role of education in shaping attitudes toward gene editing, especially regarding GE foods.

In terms of scientific literacy toward gene editing, this study first constructed a 12-item scale to objectively measure participants’ genetic knowledge levels. The findings suggest there is still potential for enhancing the general public’s understanding of gene editing, as demonstrated in Appendix A. The general public lacks clarity regarding the fundamental principles of gene editing, leading to insufficient differentiation between genetically modified organisms and genetic technologies. Given the prolonged skepticism and debate surrounding genetically modified organisms in China [20,21,116], future efforts should focus on enhancing public understanding of the distinctions between gene editing and genetically modified organism technologies through science communication. In comparison with a survey conducted in 2016 [117], there has been an increase in public knowledge about genetically modified organisms, such as the correct response rate rising from 64.2% in 2016 to 85.5% in 2022 for statements like “If consumers eat GM food, their genes can be changed”.

Moreover, the results of this study suggest that subjective and objective genetic knowledge have contradictory impacts on attitudes toward GE foods. This study found a positive correlation between subjective knowledge and WTP for GE foods, whereas objective knowledge exhibited a negative correlation with WTP for GE foods. These findings align with those reported in a prior study conducted in China. [118]. The results indicated that individuals with higher subjective knowledge perceived lower risks and higher benefits associated with gene editing technology. Conversely, higher levels of objective knowledge did not reduce perceived risks but decreased perceived benefits. This observation may be attributed to individuals with higher genetic literacy being more capable of making objective rather than overly optimistic assessments of the benefits brought about by gene editing.

Overall, previous studies generally show relatively consistent findings regarding the positive correlation between subjective knowledge and attitudes toward gene editing [31,32,55,78,108,119,120,121], although some research has not confirmed this relationship [37,122,123,124]. For instance, research has indicated a negative correlation between individuals’ subjective awareness and attitudes toward gene editing after the occurrence of the gene-edited baby incident in China [37]. Research results on the relationship between objective knowledge and attitudes toward gene editing exhibit some inconsistencies. Several studies have found a positive correlation between objective knowledge and attitudes toward gene editing technology or genetically modified organisms [64,66,69,73,74,76,125], whereas others have reported negative correlations [118,124] or no significant correlations [78,121,126]. Various potential explanations exist for these inconsistent findings. Firstly, different measurement tools in studies may produce different results. For example, some studies measured general scientific knowledge (e.g., “Which gas is produced as a consequence of burning fossil fuels?”), while others measured biological or genetic knowledge [69,78,115,127]. Previous research has shown that individuals’ diverse knowledge structures also lead to differing attitudes toward GE crops [31,75]. Secondly, the influence of individuals’ scientific literacy on attitudes toward gene editing technologies may vary across cultures or periods within the same country. For instance, cultural and experiential factors have been shown to impact public attitudes toward gene technologies [111,122]. Specifically, studies indicate that consumers with humanities and social sciences backgrounds tend to perceive more significant benefits as knowledge levels increase. In contrast, those with backgrounds in science and technology tend to perceive higher risks [128]. Following the gene-edited baby scandal in China, positive discussions about gene editing on Chinese internet platforms decreased while negative sentiments, such as disgust, increased. This suggests that despite heightened public awareness of gene editing due to the academic scandal, technology acceptance decreased accordingly [129].

**Figure A1:**
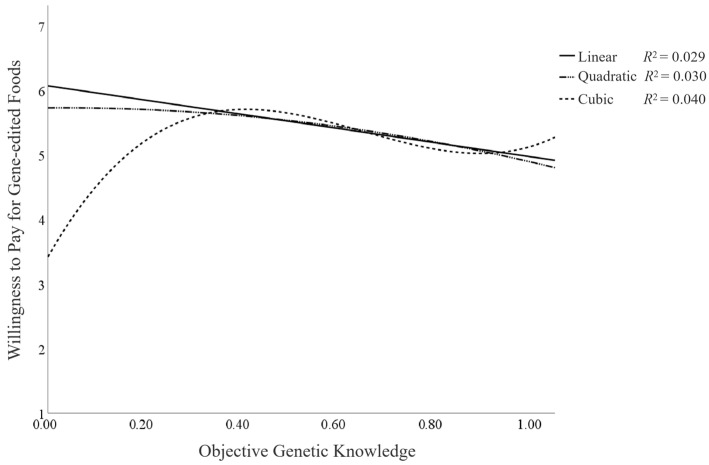
Linear and nonlinear tests of objective knowledge and WTP for GE foods.

Furthermore, the influence of scientific knowledge on attitudes towards different applications of gene editing may vary significantly. For instance, prior research has indicated that scientific literacy positively affects attitudes toward genetically modified vegetables but has no impact on attitudes toward gene-edited livestock [127]. Therefore, there are various boundary conditions between scientific knowledge and attitudes towards gene editing, with some scholars suggesting a nonlinear relationship between the two [75]. For example, Bucchi and Neresini (2002) demonstrated that more excellent information does not always lead to more positive attitudes towards GM foods [130]. To further explore whether there is a more complex nonlinear relationship between genetic knowledge and GE food attitudes, this study constructed regression models to predict WTP for GE foods using linear, quadratic, and cubic objective knowledge predictors. The results showed that the cubic regression model exhibited the best-fit index (*R*^2^ = 0.04, *p* < 0.001) compared to the linear (*R*^2^ = 0.03, *p* < 0.001) and quadratic models (*R*^2^ = 0.03, *p* < 0.001). The cubic model demonstrated a positive correlation between individuals’ genetic literacy and attitudes toward GE foods when genetic literacy was low. However, once their knowledge reaches a moderate level, their attitudes start to become more negative. This negative trend continues until they reach a high level of genetic literacy, at which point their attitudes begin to improve again (see Figure A1—Appendix B for details). This model elucidates the intricate correlation between scientific knowledge and attitudes towards GM foods. Given that this outcome was exclusively observed in the Chinese context, it is yet to be determined if this model can be applied in other cultural contexts. The cubic model indicated that people’s genetic knowledge increased, and their positive attitudes toward GE foods increased. However, after reaching a moderate level of knowledge, their attitudes toward GE foods began to trend more negatively until they reached a high level of genetic knowledge, when their attitudes started to improve again. This pattern clarified the complex relationship between scientific knowledge and attitudes towards GM foods. Since this finding was specific to the Chinese context, further investigation is needed to ascertain whether this pattern holds across diverse cultural contexts.

Scientific belief correlates with scientific knowledge but differs from it, representing a psychological belief that extends beyond knowledge structures [79]. Individuals with stronger scientific beliefs typically possess excellent scientific knowledge and exhibit more positive attitudes toward controversial technologies [68,73,74]. This study also revealed a positive association between scientific belief and attitudes towards GE foods, demonstrating that higher scientific belief correlates positively with perceived benefits and negatively with perceived risks. Previous research has shown that scientists prioritize the benefits of gene editing, while the general public tends to focus on technical risks [131]. In contrast to previous findings, our study additionally identifies a significant negative correlation between scientific belief and perceived ethical concerns, suggesting that individuals with stronger scientific beliefs may overlook technology-associated ethical issues. This underscores the necessity for enhanced ethical guidance within scientific research. Furthermore, our findings indicated a significant negative correlation between scientific belief and perceived ethical concerns, suggesting that individuals with higher scientific beliefs were more likely to ignore the ethical issues of technology. This highlighted the need for the scientific research community to strengthen the guidance of ethical norms.

Some scholars suggest that the perception of technology includes risk perception, benefit perception, and social trust perception [132,133]. The objects of social trust usually include scientists conducting gene editing research and government agencies responsible for supervision. Public trust in scientists and governments has consistently been shown to correlate closely with attitudes toward technology acceptance [43,83,87,110,134]. The findings of our study indicated that higher levels of trust in gene-editing experts and the government correspond to more positive attitudes toward GE foods. This finding aligns with previous research on GE food acceptance [30,36,87,94]. Interestingly, in this study, public trust in gene editing scientists was comparatively lower than trust in the government. This finding aligns with research on GM foods in China [117] yet contrasts with studies in Europe and the United States [87,135], where trust in scientists has been reported to surpass trust in government institutions. In fact, as a collectivist country, China has consistently maintained high levels of public trust in the government, which was clearly reflected in the extensive compliance of the public with government policies during the COVID-19 pandemic [136,137,138]. This highlights that social trust in GE foods among citizens exhibits significant internal heterogeneity across different cultures. Targeted strategies can be developed based on this framework regarding public relations and science communication. For instance, in the Chinese context, the promotion of GE foods could incorporate additional government-endorsed content or feature government certification labels during marketing.

In the past, the competence dimension of public social trust related to technology referred more to the ability of institutions and governments to understand and regulate technology [87]. This study incorporated trust in national technological capabilities within the framework of social trust, expanding the competence dimension that has been insufficiently explored in previous social trust studies.

As globalization of the economy continues, promoting cutting-edge technologies worldwide, particularly genetic technology products, often encounters complexities. Instances such as GM foods and COVID-19 vaccines have stirred conspiracy theories with nationalist undertones globally [20,139,140]. GM foods have faced significant resistance in China, accused by many of being genetic weapons wielded by other nations against the Chinese populace, a perception only recently starting to shift [141]. Previous studies at the individual level have found that lower self-confidence is associated with higher support for conspiracy theories [142]. Similarly, the GM food conspiracy theory also reflects that some people believe that the country lacks the capabilities in genetic technology to ensure the safety of foreign GM foods, leading these people to believe that foreign GM foods are biological weapons. It reflects that the combination of public perceptions of national technological prowess and nationalist sentiments can profoundly influence cutting-edge technological products. Therefore, this study measured public trust in the country’s gene editing technology capabilities from an international competition perspective. The results indicated that the higher Chinese citizens’ confidence in the country’s genetic technology prowess, the greater their perceived technological benefits and the lower their perceived risks. This suggests that, akin to trust in scientists and the government, trust in national technological capabilities can also enhance public perceptions of gene editing. However, current findings have found that this trust was significantly lower than trust in scientists and governments, posing a potential concern for the future promotion of GE foods. Failure to effectively improve public perception of the country’s genetic technology prowess may perpetuate new conspiracy theories, particularly regarding imported GE foods.

In terms of gene editing technology cognition, the theory of planned behavior posits that subjective attitudes influence behavioral intentions, which subsequently impact behaviors [143]. Therefore, individuals’ cognitive attitudes toward GE foods affect their purchasing decisions and subsequent behaviors [144,145]. The regression analysis in this study found that perceived risks and perceived ethical concerns significantly negatively predicted the WTP for GE foods. In contrast, perceived benefits positively predicted WTP for GE foods. These results align with previous research [36,73,75,94,122]. In previous studies on attitudes toward GE foods, ethical perceptions have been relatively underexplored compared to studies on public attitudes toward human gene editing [43]. In reality, the public also expresses ethical concerns about gene editing in animals and plants, although these concerns are generally less pronounced than those related to human gene editing [146]. In this study, perceptions of the ethical implications of gene editing in animals and plants were relatively low (2.69 on a 6-point scale), suggesting a relatively high ethical acceptability of gene editing technology applied to these organisms. This finding is consistent with a Canadian study indicating moderate approval of GE crops (3.39 on a 6-point scale) [122], albeit lower than the ethical acceptability perceived in our research. This highlights cultural variations in ethical perceptions of gene editing technology. Among the three categories of technological cognition investigated here, ethical perception displays distinct psychological characteristics compared to risk perception and benefit perception. Moral judgments are fundamentally different from judgments of gains and losses because moral judgments are typically non-negotiable [147]; once people perceive a technological product as unethical, acceptance becomes challenging. For example, a study conducted in the United States reported that 71% (representing 45% of the sample) of participants opposed to GM foods maintain an absolutely moral opposition, resistant to changing their views despite additional evidence. Given controversies like the gene-edited baby scandal, mitigating public perceptions of the unethical use of gene-editing technology in non-human applications is essential for fostering broader public acceptance of gene-edited products.

Regarding WTP for GE foods, this study found a generally positive attitude among participants, with a relatively high expressed WTP (5.19 on a 7-point scale) and 80.9% showing a propensity to buy (ranging from relatively desired to highly desired). Previous research has demonstrated country-specific differences in attitudes toward GE foods [36,94,148]. Compared to a study conducted in the United States, where the majority of participants opposed purchasing GM foods [30], this study discovered a more positive public preference for purchasing GE foods. Some studies have indicated that attitudes toward genetically modified and GE foods were more favorable in the United States than in France and Japan [24,148], suggesting potentially more positive attitudes toward GE foods among the Chinese population than developed countries. However, further regional studies are necessary to substantiate this claim, particularly from a broader range of countries. Prior studies on Chinese citizens have revealed that they possess a more favorable disposition towards GE foods than GM foods. Furthermore, they demonstrate a willingness to pay a higher price for GE foods [25]. It suggests that Chinese individuals hold relatively high expectations for GE foods, establishing a robust psychological foundation conducive to their widespread acceptance and actual purchase behavior in the future.

### 5.2. Theoretical Contributions and Practical Implications

This study contributes to previous research in several key aspects. Firstly, this study introduced several variables that have not been explored in quantitative studies of attitudes towards GE foods, such as belief in science, objective and subjective knowledge related to genetic engineering, confidence in national technology related to gene editing, and ethical perceptions of gene editing for animals and plants. By exploring the impact of these variables, new insights are provided for understanding the psychological causes of the public’s attitude towards GE foods. Secondly, based on the KAP model and OSOR model of behavioral intention formation [45,49], this study suggests that demographic factors, knowledge literacy, social attitudes, and gene technology perceptions progressively influence attitudes toward GE foods within the research framework, as corroborated by the study findings. For example, in Model 4 of the regression analysis, demographics and knowledge literacy variables largely lost their predictive power, indicating a potential mediation effect pattern. While this study primarily focuses on delineating the individual effects of each variable within its hierarchical context rather than examining mediation effects, it nonetheless provides new evidence for future exploration of mediation mechanisms. Furthermore, we expanded the cognition component of the knowledge–cognition–attitude–behavior model to include social trust and technical cognition (perceptions of gene editing), thereby enriching the understanding of the influence of various agents on individuals’ attitudes towards GE foods.

In terms of practical implications, Firstly, this study developed a questionnaire to measure objective genetic literacy, aimed at examining misconceptions among Chinese citizens regarding genetic knowledge and providing new insights for genetic knowledge dissemination. For example, this study found that the public’s knowledge of the differences between GE technology and GM technology is relatively weak. Therefore, it is important to strengthen popular science education on the distinctions between these two technologies. Additionally, this study revealed a complex, nonlinear relationship between the public’s genetic knowledge and their attitudes towards genetically edited foods. An increase in genetic knowledge does not necessarily lead to a more positive attitude towards genetically edited foods. Therefore, it is essential for scientists and government officials to engage in the promotion of GE foods, considering that social trust influences people’s attitudes towards GE foods. Furthermore, the study found that higher-income groups tend to have more positive attitudes towards genetically edited foods. Hence, future market promotion of genetically edited foods may consider prioritizing developed regions, as this may yield better results.

### 5.3. Limitations and Suggestions for Future Research

This study offers a systematic and hierarchical perspective for understanding the impact of various factors on the public’s attitudes toward GE foods. It is imperative to acknowledge certain limitations. Firstly, this study was conducted using a questionnaire. It lacked an experimental design to explore the causal relationships between variables. Considering the mechanism of psychological effects, studies utilizing questionnaires also employ independent variables to predict attitudes toward gene editing technology [55,87,149]. However, strictly speaking, experimental research is better suited to determine causal mechanisms in terms of methodology. In the future, researchers could consider designing corresponding experiments for such exploration.

Regarding the study sample, an online questionnaire survey may have included participants who are predominantly younger and possess higher levels of education [150]. It has partially impacted the representativeness of the study’s findings. Therefore, it is crucial for future research to use more diverse samples to accurately depict the public’s genetic knowledge and attitude toward GE foods.

This study is subject to certain limitations in the design of its research variables. For example, it did not investigate the public trust in companies that produce GE foods, nor did it quantify the actual income of individuals based on demographic factors. Subsequent studies could conduct further investigation into these variables.

In Model 4 of the regression analysis, variables related to social trust continued to exhibit significant predictive power, suggesting that perceived risks, benefits, and ethical considerations cannot fully explain these variables’ influence on attitudes toward GE foods. Therefore, future research should incorporate additional cognitive variables, such as equity perceptions [92], to further refine and expand the theoretical model. Additionally, psychological factors have also been shown to be related to attitudes towards gene editing technology, such as cultural values and an aversion to tampering with nature [74,122]. Therefore, future research should continue to explore the impact of psychological factors on attitudes towards GE foods.

## 6. Conclusions

This study employed a questionnaire survey to investigate Chinese citizens’ attitudes towards GE food and its determinants. Hierarchical regression models were utilized to examine the unique contributions of demographic factors, knowledge literacy, social attitudes, and genetic technology cognition to GE food.

The results revealed an increasing trend in people’s genetic knowledge literacy. However, compared with genetically modified knowledge, people’s knowledge of gene editing is less sufficient, revealing an imbalanced state. In addition, the study found that objective and subjective knowledge had opposite effects on GE foods. Subjective knowledge positively correlated with attitudes towards GE food, whereas objective knowledge showed an inverse relationship. Notably, the impact of objective knowledge on GE food attitudes demonstrated a significant, typical nonlinear relationship, highlighting the intricate influence of knowledge. Furthermore, individuals with higher scientific beliefs exhibited more positive attitudes toward GE foods.

Among demographic factors, this study identified that only income was positively associated with attitudes toward GE foods. Additionally, a strong relationship existed between social trust and attitudes toward GE foods. Specifically, trust in scientists, governments, and national scientific and technological capabilities all positively influenced attitudes toward GE foods. However, public confidence in national scientific and technological capabilities was notably lower compared to the former two factors, which raises concerns about the potential emergence of nationalist conspiracy theories in the field of GE foods.

In terms of gene editing technology perceptions, this study found significant correlations between risk perception, benefit perception, ethical considerations, and WTP for GE foods. Furthermore, public perceptions of gene editing technology were generally positive. People tended to perceive lower risks, higher benefits, and less ethical concern when gene editing was applied to non-human entities. Overall, attitudes toward GE foods were relatively positive, with more than 80% of respondents indicating a willingness to purchase such products.

## Figures and Tables

**Table 1:** Demographic information (*N* = 649).

Variables	Item	*N* (%)
Gender	Males	238 (36.7)
	Females	411 (63.3)
Age	<26 years	197 (29.9)
	26–35 years	344 (52.9)
	36–45 years	74 (11.4)
	>45 years	29 (4.7)
Educational level	High school or below (<13 years)	14 (2.1)
	Bachelors (13–16 years)	486 (74.9)
	Master or above (>16 years)	149 (23.0)
Income level	Lower than middle income	244 (37.7)
	Higher than middle income	405 (62.4)
Religion	Religious	69 (89.4)
	Not religious	580 (10.6)

**Table 2:** Descriptive analysis and correlation of included variables (*N* = 649).

Variables	1	2	3	4	5	6	7	8	9	10	11	12	13	14	15
1 Gender															
2 Age	0.14 **														
3 Education level	−0.02	0.13 **													
4 Religiosity	−0.03	0.11 **	−0.03												
5 Income	0.06	0.20 **	0.05	0.07											
6 Subjective knowledge	0.14 **	0.12 **	−0.04	0.13 **	0.35 **										
7 Objective knowledge	0.06	−0.07	0.18 **	−0.15 **	−0.11 **	−0.07									
8 Belief in science	0.03	0.08 *	−0.14 **	0.05	0.25 **	0.26 **	−0.25 **								
9 Trust in scientists	−0.05	−0.04	−0.01 *	0.00	0.17 **	0.20 **	−0.21 **	0.46 **							
10 Trust in government	−0.01	−0.02	−0.08 *	0.03	0.17 **	0.18 **	−0.16 **	0.44 **	0.56 **						
11 Confidence in national technology	−0.04	−0.09 *	−0.11 **	0.01	0.16 **	0.21 **	−0.20 **	0.38 **	0.49 **	0.46 **					
12 Risk perceptions	−0.03	−0.08 *	−0.02	−0.01	−0.18 **	−0.22 **	0.01	−0.28 **	−0.24 **	−0.22 **	−0.24 **				
13 Benefit perceptions	0.05	0.10 **	−0.05	0.11 **	0.22 **	0.24 **	−0.20 **	0.46 **	0.46 **	0.45 **	0.36 **	−0.47 **			
14 Ethical perceptions	−0.02	−0.30	0.11 **	0.36	−0.15 **	−0.14 *	0.21 **	−0.21 **	−0.18 **	−0.20 **	−0.22 **	0.31 **	−0.31 **		
15 Attitudes	0.04	−0.02	−0.07	0.07	0.19 **	0.23 **	−0.17 **	0.40 **	0.50 **	0.50 **	0.46 **	−0.41 **	0.56 **	−0.30 **	
M	0.37	30.21	16.42	0.11	5.75	4.41	0.67	4.99	5.72	5.8	4.07	4.15	5.24	2.69	5.19
SD	0.48	8.20	2.09	0.31	1.29	1.25	0.19	1.09	1.12	1.12	0.86	1.28	1.11	1.36	1.16

Note. * *p* < 0.05; ** *p* < 0.01.

**Table 3:** Hierarchical regression analysis of attitudes towards GE foods.

	Model 1		Model 2		Model 3		Model 4	
Variables	*β*	*p*	*β*	*p*	*β*	*p*	*β*	*p*
*Demographics*								
Gender	0.04	0.28	0.03	0.38	0.06	0.09	0.05	0.11
Age	−0.06	0.11	−0.09 *	0.02	−0.04	0.32	−0.07 *	0.03
Education level	−0.07	0.09	0.07	0.85	0.02	0.631	0.01	0.75
Religiosity	0.06	0.11	0.03	0.35	0.05	0.15	0.03	0.30
Income	0.20 **	<0.01	0.07	0.09	0.04	0.32	0.01	0.72
*Scientific literacy and beliefs*								
Subjective knowledge			0.12 **	<0.01	0.07	0.06	0.04	0.24
Objective knowledge			−0.08 *	0.048	−0.03	0.45	−0.01	0.76
Belief in science			0.34 **	<0.01	0.11 **	<0.01	0.01	0.71
*Social trust*								
Trust in scientist					0.22 **	<0.01	0.15 **	<0.01
Trust in government					0.25 **	<0.01	0.19 **	<0.01
Trust in national technology					0.14 **	<0.01	0.09 *	0.02
*Perceptions of gene editing*								
Risk perceptions							−0.15 **	<0.01
Benefit perceptions							0.24 **	<0.01
Ethical perceptions							−0.10 **	<0.01
*R-square*	0.05 **		0.20 **		0.37 **		0.46 **	
*ΔR-square*	——		0.15 **		0.16 **		0.10 **	

Note. * *p* < 0.05; ** *p* < 0.01.

## Data Availability

The datasets are not publicly available. The data presented in this study are available on request from the corresponding author due to at the questionnaire stage, we promised the respondents that the data would be kept confidential, therefore, we cannot make the data public.

## Appendix A

**Table A1:** Genetic knowledge questions.

Domains	Questions	Raw Responses from Participants (%)	
		Wrong	Right
Genetic modification	Traditional foods do not contain genes, but genetically modified foods contain genes.	82.9%	17.1%
	If consumers eat GM food, their own genes can be changed	85.5%	14.5%
	Genetically modified animals are always larger than ordinary animals.	76.4%	23.6%
Gene editing	You can tell whether a fruit has been gene-edited by its appearance.	79.2%	20.8%
	The principle of gene editing is making genes that initially had no function functional.	35.5%	64.7%
	Gene editing will not have side effects due to off-target effects.	67.2%	32.8%
	If fish genes are inserted into tomato, tomatoes will have fish flavor	61.2%	38.8%
	Gene editing has the potential to expedite the recovery of fracture patients within a few days.	76.6%	23.4%
Distinction between GM and GE	Both genetic modification and gene editing can add corn genes to rice.	31.1%	68.9%
	If you want to make tomatoes taste like corn, it can only be achieved through genetic modification.	54.2%	45.8%
	Both genetic modification and gene editing can have side effects, rendering the foods produced by these technologies unsuitable for eating.	92.1%	7.9%
	Herbicide-resistant rice can only be obtained through genetic modification technology	57.6%	42.4%
